# How much can we gain from improved efficiency? An examination of performance of national HIV/AIDS programs and its determinants in low- and middle-income countries

**DOI:** 10.1186/1472-6963-12-74

**Published:** 2012-03-24

**Authors:** Wu Zeng, Donald S Shepard, Jon Chilingerian, Carlos Avila-Figueroa

**Affiliations:** 1 Schneider Institutes for Health Policy, Heller School, MS 035, Brandeis University, Waltham, MA 02454-9110, USA; 2 Abt Associates, 4550 Montgomery Ave., Suite 800 North, Bethesda, MD 20814, USA

**Keywords:** HIV/AIDS, Performance, Efficiency, Governance, Data Envelopment Analysis

## Abstract

**Background:**

The economic downturn exacerbates the inadequacy of resources for combating the worldwide HIV/AIDS pandemic and amplifies the need to improve the efficiency of HIV/AIDS programs.

**Methods:**

We used data envelopment analysis (DEA) to evaluate efficiency of national HIV/AIDS programs in transforming funding into services and implemented a Tobit model to identify determinants of the efficiency in 68 low- and middle-income countries. We considered the change from the lowest quartile to the average value of a variable a "notable" increase.

**Results:**

Overall, the average efficiency in implementing HIV/AIDS programs was moderate (49.8%). Program efficiency varied enormously among countries with means by quartile of efficiency of 13.0%, 36.4%, 54.4% and 96.5%. A country's governance, financing mechanisms, and economic and demographic characteristics influence the program efficiency. For example, if countries achieved a notable increase in "voice and accountability" (e.g., greater participation of civil society in policy making), the efficiency of their HIV/AIDS programs would increase by 40.8%. For countries in the lowest quartile of per capita gross national income (GNI), a notable increase in per capita GNI would increase the efficiency of AIDS programs by 45.0%.

**Conclusions:**

There may be substantial opportunity for improving the efficiency of AIDS services, by providing more services with existing resources. Actions beyond the health sector could be important factors affecting HIV/AIDS service delivery.

## Background

Since the beginning of the epidemic of Human Immunodeficiency Virus/Acquired Immune Deficiency Syndrome (HIV/AIDS), almost 60 million people have been infected with HIV and 25 million people have died of HIV-related causes [1]. Despite the billions of dollars spent on this disease, a gap persists between resource needs and resources available for HIV/AIDS control and treatment [2,3]. The economic downturn since 2008 exacerbates the inadequacy of resources for combating the worldwide HIV/AIDS pandemic [4]. These resource constraints amplify the need to improve the efficiency of HIV/AIDS programs [5].

Efficiency studies conducted at the organizational level (e.g., health centers) show a substantial variation of performance (measured by program unit cost) of HIV/AIDS interventions both within a country and across countries [6-9]. While some of the variation is attributable to differences in the scale and the breadth of each country's AIDS interventions, much remains unexplained. Anecdotal and qualitative reports contend that the inefficiencies of HIV/AIDS programs at a national level are largely due to high transaction costs, repeated investment, poor government responsiveness, and low level of human resource skills [10-12]. However, these factors are poorly documented with quantitative empirical data. Examining the impact of these factors would strengthen policies and resource allocation for scaling up HIV/AIDS services. For example, in countries where the efficiency of HIV/AIDS services is low, priority should be given to interventions to overcome barriers against implementing efficient programs. On the other hand, in countries where performance is already high, efforts should be geared towards mobilizing more resources.

This study uses comprehensive data released in 2008 by the Joint United Nations Programme on HIV/AIDS (UNAIDS) [13] to quantitatively evaluate efficiency of countries' use of funding for HIV/AIDS, and then to explore the impact of some social and economic determinants on the efficiency.

## Methods

This study followed a classical framework of economic analysis of efficiency using a two-step process: in the first step efficiency of national HIV/AIDS programs were evaluated with direct inputs and outputs, and in the second step econometric models were applied to explain the efficiency [14]. Thus this study considers three sets of variables from each country: (1) direct inputs of national HIV/AIDS programs, (2) direct outputs of the programs, and (3) contextual factors affecting the efficiency.

We used the data from UNAIDS reports on national AIDS spending to determine the inputs for national HIV/AIDS programs [13,15]. We standardized expenditures into 2007 international dollars (I$) after adjusting for purchasing power parity (PPP) and inflation. We selected the number of people receiving voluntary counseling and testing (VCT), the number of HIV+ pregnant women receiving HIV/AIDS treatment for prevention of mother-to-child transmission (PMTCT), and the number of patients receiving antiretroviral treatment (ART) as program outputs. These three indicators were selected as outputs because they are the standardized services that could be compared across countries, and they are the services for which the data are widely available and are measured more precisely. One may argue that using these three indicators as outputs may result in unfavorable results for those countries that focus on prevention outside of health facilities. Based on the data on AIDS spending released by UNAIDS in 2009 [14], we found that VCT and PMTCT were good proxies for AIDS prevention activities; together they were a major component of prevention activities, accounting for 40.3% of total spending on prevention, and spending on VCT and PMTCT was highly correlated with expenditure on prevention with a correlation coefficient of 0.67 (p < 0.001).

Four aspects have been highlighted by the World Bank to improve the efficiency of funding, which include 1) macroeconomic status, 2) social and cultural factors, 3) infrastructure and human resources, and 4) institutional and policy environment [4]. From the available data, we included indicators on economic and demographic characteristics, health financing mechanisms, and governance as key potential determinants of the unconditional technical efficiency in the regression analysis. Additional file 1 provides a detailed description of the selection of the inputs, outputs and determinants.

Data on national HIV/AIDS expenditure and the three types of HIV/AIDS services (VCT, PMTCT and ART) were obtained from UNAIDS documents [13,16-19]. After compiling them, we imputed the missing values. For countries with data available for at least two years, we interpolated or extrapolated missing values using log linear function. For countries with data available for one year only, regional growth rates of the services or spending were used to impute missing values. To check the consistency and validity of imputed data, we examined the distribution of the imputed data and performed separate regressions with observed and imputed data of VCT on ART and ART on AIDS spending. We found that 98% of imputed data points fell within the 95% confidence interval of observed data and there were no statistically significant differences of the slopes in the corresponding regressions [20,21]. Data on potential determinants were obtained from databases constructed by the World Bank and the World Health Organization [22,23]. The final data set covers 68 countries with 151 observations, spanning the years 2002 through 2007.

We used output-oriented data envelopment analysis (DEA), a classic non-parametric approach to evaluating technical efficiency [24], to estimate the efficiency of national HIV/AIDS programs. The conventional Charnes, Cooper, and Rhodes (CCR) model is described further in Additional file 1[24]. As the CCR model does not incorporate prior knowledge of the relative importance of inputs and outputs in estimating weights [25], we used the DEA model with an assurance region (AR), setting weight boundaries for outputs so that the relative importance of each of the services contributing to the efficiency is within an appropriate range; these are described in Additional file 1.

We used DEA-solver 5.0 (Saitech Inc. New Jersey) to estimate two sets of efficiency scores for each observation using DEA models. The first set was obtained by running the DEA model on separate years (each country was compared to its peers in the same year) to estimate the efficiency of countries in implementing HIV/AIDS programs (hereafter termed 'separate DEA'). The second set was calculated by pooling all countries over years with one DEA model (each country was compared to the best performers among the six years) (hereafter termed 'pooled DEA'). The results from pooled DEA were used to examine the evolution of the performance of national HIV/AIDS programs over time and to conduct the regression analysis, as described below. As these efficiency scores were not adjusted for any factors, we termed them unconditional efficiency scores.

After obtaining the efficiency scores from the pooled DEA model, we constructed a random-effects Tobit regression model with finite population adjustment as the second stage of the efficiency analysis [14,26,27]. To generate consistent estimates in the second stage analysis, it requires the independency of inputs from contextual factors used [26]. We examined the association between HIV spending and the contextual factors used in the Tobit model and found a low level of correlation between them with R^2 ^= 0.20, which permits the use of Tobit model in the study. The Tobit model was built on 120 observations through 2006 because the information on health financing, one of the independent variables, was available only through that year at the time of the analysis (2009). We adjusted the unconditional efficiency scores for those factors, obtaining the conditional efficiency of a country's national AIDS program. We defined a "notable increase" as the change from the lowest quartile to the average value of a variable. Using the Tobit model, we derived the improvement in unconditional efficiency of an AIDS program from "notable" increases in all key determinants. The second stage analysis was conducted with STATA 11.0 (StataCorp LP, Texas)

## Results

Of all 151 observations, 18% (27 out of 151) were on the production frontier with efficiency scores of 100% using the separate DEA models. The high performers included countries that have been internationally recognized for their successful HIV/AIDS programs such as Thailand (2004, 2006, and 2007), Brazil (2005), and Rwanda (2003). The concordance of our results with empirical observations helps validate our application of DEA to national HIV/AIDS programs and the selection of inputs and outputs. Our highly efficient performers also included some countries that have received less recognition, such as China (2003 and 2006), Chad (2004 and 2006), South Africa (2005, 2006, and 2007) and Paraguay (2006 and 2007). These countries were repeatedly ranked with 100% efficiency. Complete efficiency scores from the DEA models for each country are provided in Additional file 1.

The performance of countries' HIV/AIDS programs varied widely. The mean efficiency scores by quartile across 151 observations over the six years were 13.0% in quartile 1 (the worst fourth of observations), 36.4% in quartile 2, 54.4% in quartile 3, and 96.5% in quartile 4 (the best fourth of observations). The overall average output-oriented efficiency was 49.8% (SD = 31.2%), indicating that the countries, on average, could have doubled their outputs if they used money as efficiently as their comparison peers in the same year. When concentrating on inefficient countries alone, the average efficiency score was 38.6% (SD = 23.7%), suggesting a possibility of increases outputs by 2.6 times if they performed well.

**Figure 1:**
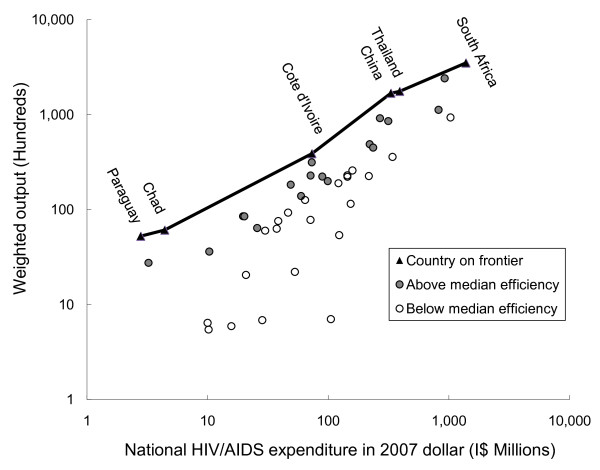
**Reduced two-dimensional display of the performance for 45 countries in 2006**. Source: Authors' analysis of data compiled from 45 developing countries in 2006. Note: These efficiency results came from a separate DEA run for data in 2006 only.

To illustrate DEA more intuitively, Figure 1 approximates the DEA model using 2006 data, containing 45 observations, with a two-dimensional graph reduced from an original four-dimensional process (one input and three outputs) by standardizing the weight for PMTCT (0.10) and VCT (0.01) relative to ART (1.0). For the ease of interpretation we arbitrarily set the weight for ART as 1, and computed the standardized weights for PMTCT and VCT by averaging the relative weights for PMTCT and VCT generated from the output-oriented DEA model across the 45 observations. The standardized weight approximately represents the relative cost for each service. One output unit was defined as the equivalent of providing ART for 100 patients for one year.

**Figure 2:**
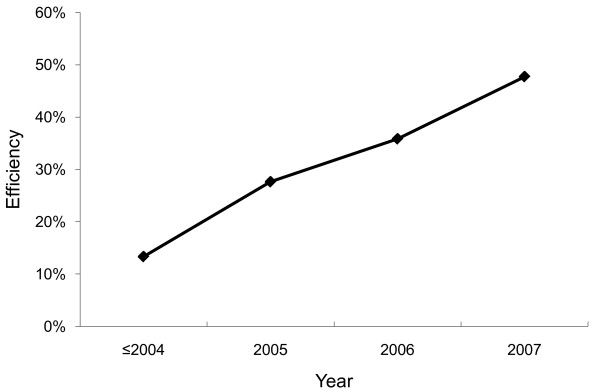
**Change in efficiency of national HIV/AIDS programs in the period of 2002-2007**. Source: Authors' analysis of data compiled from 68 developing countries from 2002 to 2007. Notes: The efficiency was evaluated by the pooled DEA. Each point represents the mean efficiency in the year(s). Due to the small sample size in years 2002 through 2004, we summarized the efficiency scores in those years.

As expected, the results from the pooled DEA model exhibited a wider variation of national HIV/AIDS programs. Eight observations fell on the production frontier with efficiency scores of 100%. They were Thailand (2007), South Africa (2007), Paraguay (2006, 2007), China (2006), Peru (2007) and Chad (2004, 2006). It was not surprising that the observations on the production frontier were those from recent years because productivity has generally increased over time. Figure 2 shows that efficiency of HIV/AIDS programs improved over time from 13.3% before through 2004 to 47.7% in 2007.

Table 1 shows the coefficients estimated from the random-effects Tobit model. Of all the factors on governance, "voice and accountability" and "government effectiveness" were found to be beneficial in improving the efficiency of HIV/AIDS programs. If the countries achieved a notable increase in "voice and accountability" (from -1.37 to -0.33), the efficiency would increase by 40.8%.

**Table 1:** Determinants of efficiency of national HIV/AIDS programs

Determinant (independent variable)	Coefficient	Standard Error
Voice and accountability (VA)**	0.392	0.208
Government effectiveness (GE)	0.500	0.488
Rule of law (RL)	-0.333	0.475
Control of corruption (CC)	-0.323	0.428
Government commitment to health*	0.031	0.021
External source as percentage of total health expenditure **	0.030	0.014
Government source as percentage of total health expenditure *	-0.014	0.008
CC × RL **	0.602	0.312
CC × external share of health spending*	0.022	0.012
Log(GNI per capita)*	3.485	1.992
Square of log(GNI per capita)*	-0.207	0.128
Log(adult population)*	0.147	0.091
HIV/AIDS prevalence (in percentage points)**	0.052	0.021

Source: Authors' analysis of determinants of the efficiency of national HIV/AIDS programs, based on the efficiency scores generated from the pooled DEA model. Notation: * p < 0.05, ** p < 0.01. Notes: The dependent variable is natural logarithm of efficiency scores from output-oriented DEA over years; coefficients for dummy variables on years 2003-2007 and the constant are suppressed for brevity; log refers to natural log; the total number of observations is 120. GNI denotes gross national income. The variance of the random error is estimated at 0.111. Additional file 1 (Table A5) provides the full model specification.

The positive coefficients for the two interaction terms in the model indicate that fighting corruption would significantly improve the efficiency of using external funds and strengthen the impact of policy implementation on delivering HIV/AIDS services.

Our findings also show that a higher share of expenditure from external sources generally improved the efficiency of HIV/AIDS programs. Greater external shares had a higher impact on efficiency in a country with lower levels of corruption. For countries with mid-level efforts in "control of corruption" (value is 0), a notable increase in the external share of health expenditure (from 1.0% to 5.5%) would result in a 13.5% increase of efficiency.

The random-effects Tobit model shows that the relationship between per capita gross national income (GNI) and efficiency followed an inverted U-shape. Efficiency rose with increasing GNI/capita up to the level of I$ 4468. At that level, which corresponds to the income of lower- and middle-income countries in Europe, Central Asia, Latin America and the Caribbean [28], efficiency was maximized. The income elasticity of efficiency for a country at the median level of income (I$2490) in the sample was 0.25. In other words, such a country could increase its efficiency in implementing HIV/AIDS programs by 1% with a 4% increase of its GNI/capita. A notable increase in GNI/capita (from I$ 1135 to I$ 2490) would raise the efficiency of AIDS programs by 45%.

## Discussion

A 2009 analysis by UNAIDS showed that donors' financial commitments to HIV/AIDS, while impressive, cover only 54% of the resources needed to meet the goal of universal access [2]. At the AIDS 2010 conference, analysts warned that weak economic conditions jeopardized continued funding for HIV/AIDS. Most analyses conclude that improving the efficiency of existing investments would contribute to resolving this challenge.

We found that the unconditional efficiency of national HIV/AIDS programs was about 50%. This result echoes the previously reported wide variation of efficiency in delivering selected HIV/AIDS services [6]. We note, however, that i) these unconditional efficiency scores do not account for the countries' characteristics (e.g., prevalence of HIV), and thus the scores may not perfectly match experts' perception; ii) the non-parametric DEA does not account for measurement errors, although considered in the second stage of the DEA model. Poor countries are likely to have lower accuracy in term of measuring both inputs and outputs; and iii) for countries that achieved 100% efficiency, the result does not mean that their efficiency cannot be improved in the future; it simply says that at a given level of expenditure on AIDS no other countries produce more outputs than they do. Their performance could be improved through enhanced procurement, better management of human resources, and other advances of management and technical interventions.

If all the countries studied were completely efficient, taking all 151 observations as a whole, their output levels would have increased by 112% for VCT, 65% for PMTCT, and 85% for ARV treatment (see Table A4 in Additional file 1). The 27 efficient observations would, at least, need to maintain service outputs at the historical level of expenditures. For the 124 inefficient observations, however, the volume of services would need to increase by 198% for VCT, 128% for PMTCT, and 155% for ARV treatment at current spending levels. These projected increases might raise VCT services in some countries beyond their needs as estimated by UNAIDS. To avoid that excess, countries could alternate PMTCT and ARV treatment services instead if funding were allocated and used wisely. Alternatively, countries could substitute regular VCT for an intensive level of VCT with better quality to enhance the effectiveness of VCT on averting risk populations from HIV infection [14].

The major contribution of this study is that it provides further insights into global resource allocation for HIV/AIDS. Findings indicate not only adjustments to total donor funding for a country, but also constructive changes in country infrastructure. To expand the coverage of HIV/AIDS services, countries could seek to obtain more resources, improve efficiency, or pursue a combined approach. Countries such as Thailand, China, and Brazil which have been efficient in providing services would need to increase financial support in order to scale up HIV/AIDS services. For countries with lower efficiency but a high HIV/AIDS burden, a combined approach is needed to simultaneously strengthen the capacity of public systems to increase efficiency for the long-term sustainability of AIDS control, and improve resource allocation to address immediate needs. In addition, by enhancing the efficiency with which HIV/AIDS funding is used, countries with low or moderate HIV/AIDS burden could reduce their HIV/AIDS spending and reallocate their limited resources to other diseases [29]. Table 2 categorizes the countries in our 2006 analysis based on: 1) the efficiency of their HIV/AIDS program and 2) the type of HIV/AIDS epidemic.

**Table 2:** A two-dimensional categorization of 45 countries in 2006

Service scale-up mechanism	**Type of HIV/AIDS epidemic**	
	**Low or concentrated (25 countries)**	**Generalized (20countries)**

Resource oriented (22 countries)	Brazil, China, Jamaica, Mali, Mauritius, Paraguay, Peru, Thailand, Uruguay	Botswana, Cameroon, Chad, Congo Dem. Rep., Cote d'Ivoire, Lesotho, Mozambique, Rwanda, South Africa, Swaziland, Togo, Uganda, Zambia

Efficiency oriented (23 countries)	Argentina, Belize, Cambodia, Colombia, El Salvador, Guatemala, Honduras, Indonesia, Iran, Lao RDR, Latvia, Nepal, Niger, Romania, Senegal, Vietnam	Angola, Benin, Burkina Faso, Central African Republic, Eritrea, Haiti, Tanzania

Source: Authors' analysis of data compiled from 45 developing countries in 2006

Notes: The HIV/AIDS epidemics are classified according to WHO.^a ^We categorized countries according to the efficiency of their national AIDS programs using a separate DEA. for data in 2006 only. Countries above the median efficiency were termed "resource oriented" while those at or below the median were termed "efficiency oriented"

Improving governance of a country is a long-term process but it brings sustained and substantial benefits. In our analysis, high transparency, accountability and involvement of local communities in decision-making are major contributors to improved efficiency of HIV/AIDS programs. The results are consistent with the participatory involvement of civil society in the control of HIV/AIDS advocated by UNAIDS and WHO [30]. Strengthening civil society through voice and accountability allows civil society to be more active in engaging in making policy in favor of vulnerable populations (e.g. the poor and people with HIV/AIDS). In many circumstances, civil society is directly involved in service delivery (e.g. medical treatment, HIV/AIDS testing, and disaster relief) to needed populations to complement governments' efforts, ensuring the services meet the needs of target populations [31,32]. Please note that our study does not measure "voice and accountability" specific for AIDS. The close relationship between "voice and accountability" in a country and HIV/AIDS service delivery indicates that the improved HIV/AIDS service provision are probably due to active involvement of HIV/AIDS civil society where "voice and accountability" for AIDS are high too. For the over 50% of all observations with a negative score on "voice and accountability," there is great potential for improving HIV/AIDS service delivery.

The inverted U-shape relationship between GNI/capita and efficiency in the random-effects Tobit model suggests a positive association between GNI/capita and AIDS program efficiency among countries with GNI/capita < I$ 4468. This association is probably due to better general infrastructure (e.g., road and transportation) and higher health capacities (e.g., personnel, information system, coordination, and management) among countries with higher per capita income, which increases both the demand for and the supply of AIDS services and thus contributes to the high volume of services being provided at lower costs (economies of scale). However, the association between GNI/capita and the efficiency is opposite among countries with GNI/capita > I$ 4468 where, usually, health infrastructure has been in place. Often wages for health personnel and the expected quality of services is higher among wealthier countries [33,34]. These factors would result in a higher unit cost for HIV/AIDS service provision in those countries.

Concerns have been raised as to how best to target the limited resources to poor countries with greater needs for HIV/AIDS [32]. As a supplemental analysis, we examined the association between GNI/capita with the efficiency scores using a pooled Tobit model. Unlike the random-effects Tobit model that takes a country-specific effect into consideration, the pooled Tobit model excludes it because the country-specific term may reflect unobserved implementation capacity of HIV/AIDS programs in that country. We found there was no relationship between the efficiency of HIV/AIDS programs and GNI/capita (t = 0.30 for log[GNI per capita] and t = -0.11for square of log[GNI per capita], both p > 0.05), which suggests that low-income countries could be at least as efficient as wealthier countries, and that the poor and wealthier countries have similar levels of performance in implementing HIV/AIDS programs as long as they have comparable policy and institutional development [35]. This finding rebuts the argument that donors should reduce funding for HIV/AIDS services in low-income countries. International donors instead need to continue their efforts in poor countries, helping them shape health care systems and improve their governance to address HIV/AIDS. It is beneficial, not just from an ethical perspective, to boost efforts to combat HIV/AIDS in the poorest countries. From a technical perspective, investing in low-income countries does not reduce the pace of the global battle with HIV/AIDS.

The main results of this study measure the unconditional efficiency of a country overall in addressing HIV/AIDS, but do not necessarily reflect the performance of the country's health care system specifically, which is often operated under the constraints of a country's general contextual factor (such as the type of epidemics and governance).

Critics may question the use of a single production frontier to evaluate all the countries in all years, since countries face different types of epidemics and different political constraints. For example, some countries may perform well because of the favorable environment, such as better governance, while other countries may deliver a mediocre amount of HIV/AIDS services under a harsh environment. When evaluating the efficiency of health care systems in addressing HIV/AIDS, we have to take into consideration countries' environmental factors beyond health sectors by adjusting the efficiency of HIV/AIDS programs for them. The adjustment is analogous to generating multiple production frontiers for the countries to be evaluated. We used the coefficients in Table 1 to estimate the efficiency of the health care system at the mean level of countries' factors. We included all the independent variables in the regression except the three variables on health expenditure that fall within the health care system. For a country with favorable contextual factors, the adjustment lowered the estimated efficiency, since some reasons for success fell outside of the health system. Conversely, the efficiency was adjusted up if a country has a harsher policy environment. For example, the efficiency of Brazil (2006) increased to 100% after the adjustment. Country-level results are available from the authors.

As evidence for the validating the use of DEA to assess the efficiency of national HIV/AIDS programs, we found that 1) some countries (e.g., Brazil, Rwanda, and Thailand), whose performance is widely respected, received excellent unconditional scores from our analysis; more countries emerge as conditionally efficient after the adjustment for the epidemics and macroeconomic factors, and 2) most coefficients from the Tobit regression bear the expected signs.

Our findings are subject to several caveats, however, and should be interpreted with caution. First, the selection of inputs and outputs was limited by data availability. Due to this constraint, we could not include human resources as an input and some key services, such as support to orphans and vulnerable children, as outputs in the model. Furthermore, as all three outputs (VCT, PMTCT and ARV) are services in health facilities [36], the unconditional efficiency scores favor countries focusing on medical activities. South Africa's high unconditional efficiency score is an example. Further refinement of efficiency scores could be examined using the country's HIV/AIDS prevalence and results of Tobit regressions. Second, the assumption that the outputs for the year result entirely from inputs in the same year in the DEA model is not completely valid in HIV/AIDS programs. Lags between inputs and outputs exceeding one year exist in many international assistance programs [37]. Third, the quality of the outputs is not factored into the analysis due to the absence of systematic data. More limited studies have shown variations in the effectiveness of a given intervention across countries [21,37], indicating the value of monitoring and evaluating the quality of services in a country's HIV/AIDS program. If a trade-off between quantity and quality exists, we should regard the coefficients in the model as upper bound of the estimates given the positive association between governance and the quality. Fourth, the country-level data may have a serious measurement problem. It is likely that measurement errors are not independent from the contextual factors (e.g. countries with poor governance are more likely to have measurement errors). The presence of measurement errors could bias the estimates for the contextual factors. Given these limitations, we note that our results are indicative rather than conclusive.

As this study concentrated on the technical efficiency of service delivery, we acknowledge that the efficiency of a country's HIV/AIDS program must be interpreted in a broader context of that country, because efficiency is not the only one domain in evaluating a program. Other criteria concern overall effectiveness, as well as economic, political and equity issues.

Two future studies would merit special attention. First, a similar study could be expanded to include additional indicators, which might focus specifically on characteristics of the health system rather than the overall society. The current study has shown the feasibility and value of that approach. Second, as efficiency can substantially relieve the financial pressure for scaling up HIV/AIDS services, it would be important to incorporate it into future projections of resource needs for HIV/AIDS.

## Conclusions

Although efficiency scores of national HIV/AIDS programs are only indicative, given the limitations of the study, our findings suggest that there may be substantial room for improving HIV/AIDS services at the country level with the existing resources. Improving the efficiency of HIV/AIDS services requires actions not only within the health sector, but also in the broader context, such as governance, to ensure that HIV/AIDS funding materializes into services that are targeted to and benefit the population in need. Given the limited resources available for HIV/AIDS services, it is critical to understand countries' performance in delivering HIV/AIDS services to inform strategies to scale up HIV/AIDS services and to help allocate the available funding wisely to combat HIV/AIDS.

## Endnote

^a^WHO, UNAIDS, UNICEF. Towards universal access: scaling up priority HIV/AIDS interventions in the health sector: progress report. 2007.

## Competing interests

The authors declare that they have no competing interests.

## Authors' contributions

All authors have made substantial contributions to the intellectual content of the paper. WZ contributed to the conception and design, acquired the data, analyzed and interpreted the data, and drafted and revised the manuscript. DSS contributed to the conception and design of the study, acquired the data, interpreted the data, and critically revised the manuscript. JC contributed to the critical revision of the manuscript for important intellectual content, and provided technical support. CA contributed to the acquisition of data, interpretation of data, obtaining funding, and providing technical support. All authors read and approved the final manuscript.

## Pre-publication history

The pre-publication history for this paper can be accessed here:

http://www.biomedcentral.com/1472-6963/12/74/prepub

## Supplementary Material

Additional file 1 **contains additional references **[38-44]. Click here for file
