# Supplementary material for: How much can we gain from improved efficiency? An examination of performance of national HIV/AIDS programs and its determinants in low- and middle-income countries
Source: BMC Health Serv Res. 2012 Mar 24;12:74. doi: 10.1186/1472-6963-12-74 (PMC3353196; doi:10.1186/1472-6963-12-74)
Supplement: Additional file 1 — contains additional references [38-44]. [file 1472-6963-12-74-S1.DOC]

**Web-appendix 1. Selection of inputs, outputs and candidate determinants**

**Inputs for DEA model (national HIV/AIDS expenditure)**. An ideal input for applying DEA to national HIV/AIDS programs would be each country’s total national expenditure on HIV/AIDS. This total should capture the cost of prevention, treatment, support for orphans and vulnerable children (OVC) and program support costs, including training human resources, administration and surveillance, and all other activities and dimensions contained in the UNAIDS resource needs model.[38] Available data that most closely match these criteria are the National AIDS Spending Assessments (NASA) compiled by UNAIDS, which are used in this study. Those data are reported in current US dollars in the year to which they refer. To make the spending estimates comparable among countries and across time, we converted them into values for 2007 international dollars (I$) by adjusting for purchasing power parity (PPP) and inflation. We used the formula:

Spending in data year (in 2007 I$) = [spending in data year (in current US$)] x [GDP deflator from data year to 2007] x [local currency units per US dollar in data year at market exchange rate] / [local currency units per US dollar in data year at PPP exchange rate].

As indicated by classical production theory, production inputs often include capital and human resources. In this study, total national HIV/AIDS expenditure could not fully capture the human resources utilized for HIV/AIDS services. We would have liked to include data from each country on the number of health personnel working in HIV/AIDS or the entire health system. However, available data for low and middle-income countries were very incomplete, and therefore infeasible to include. Also, the total HIV/AIDS expenditure, as our solo input, could be criticized because it combines resources for current use with those with a longer return such as training health personnel and building infrastructure. The mismatched time frame between the expenditure and the returns of the expenses could bias the evaluation. As both short and long term needs are associated with the demand for HIV/AIDS services in a country, we partially addressed this criticism by including HIV/AIDS prevalence as one of determinants of the efficiency in the Tobit regression.

**Defined outputs for DEA model (HIV/AIDS services)**. The ideal output indicators would be all HIV/AIDS related health and social services produced by the national program. We selected outputs for our analysis based on the criteria that the indicators should be representative and important to HIV/AIDS control, reliably measured, and available for a large number of countries. Three indicators met these criteria: 1) number of people receiving voluntary counseling and testing (VCT), 2) number of HIV+ pregnant women receiving HIV/AIDS treatment for prevention of mother-to-child transmission (PMTCT), and 3) average number of patients receiving antiretroviral treatment (ARV). These services are considered as essential services for fighting against HIV/AIDS. We have also considered including services for OVC and administrative services as outputs. Again, data availability is the major barrier that precluded their inclusion in the DEA models.

**Potential determinants of the efficiency.** Four aspects have been highlighted by the World Bank to improve the efficiency of funding, which includes 1) macroeconomics, 2) social and cultural factors, 3) infrastructure and human resources, and 4) institutional and policy environment. We included several sets of candidates to examine their impact on the efficiency of national HIV/AIDS programs: governance, financing mechanisms, and economic and demographic characteristics. In this study, the variables on governance are: 1) voice and accountability, measuring “the degree to which citizens participating in government affairs, and freedom of expression, freedom of association and a free media;” 2) government effectiveness, measuring “the quality of public services, the quality of the civil service…the quality of policy formulation and implementation;” 3) rule of law, measuring “the extent to which agents have confidence in and abide by the rules of society” 4) control of corruption, measuring “the extent to which public power is exercised for private gain;” and 5) commitment to health and HIV/AIDS, measured by the share of general government health spending out of total central government expenditure. For the first four indicators, scores range from -2.5 to +2.5, where higher scores indicate better performance. These indicators have been used widely in econometric models. The variables regarding financing mechanisms are: 1) external resources for health as a percentage of total expenditure on health, and 2) general government expenditure on health as percentage of total expenditure on health. [23] The economic and demographic variables include 1) Gross National Income (GNI) per capita in PPP, 2) HIV/AIDS prevalence for ages 15 to 49, and 3) the adult population (ages 15-49) in the country.[41] We also included the year dummies in the model to control for the time fixed effects, such as the secular change of the price of ARV drugs.

# Web-appendix 2. Basics on data envelopment analysis (DEA)

Data envelopment analysis (DEA), developed in 1978, has been applied and has flourished in the past 30 years to evaluation the technical efficiency of decision-making units in various settings. Here we provide concise introduction of this method, and for in-depth discussion, see its original work and relevant books

The idea of DEA is similar to frontier analysis. DEA first identifies the best performers and then compares the remaining performers to the best. Unlike many methods that can examine only one input and one output at a time, DEA can manage the complexity of multiple inputs and multiple outputs simultaneously. The ratio of the combined inputs and outputs is regarded as the efficiency score.

Intuitively, we would like to measure the technical efficiency (TE) by taking the ratio of the outputs (y) to the inputs (x), as showed in equation (1) below.

$TE=\frac{y_{1}+y_{2}+\cdots+y_{r}}{x_{1}+x_{2}+\cdots+x_{i}}=\frac{1}{\frac{x_{1}+x_{2}+\cdots+x_{i}}{y_{1}+y_{2}+\cdots+y_{r}}}$ (1)

where *y_r_* is the amount for the output *r*, and *x_i_* is the amount for the input *i*.

However, because the inputs and the outputs are usually measured on different scales, we cannot add them directly. We could overcome this problem if we could multiply each input and output by an appropriate weighting factor. Unfortunately, we do not know the weights associated with inputs and outputs in most cases. Technical efficiency is the inverse of the ratio of inputs to outputs.

DEA can help us derive these unknown weights. Here we examine the inverse of the TE rather than TE itself. It minimizes the ratio of the single virtual input to a single virtual output (which is function of the multipliers) by requiring that the ratio should be greater than 1 to obtain the efficiency score between 0 and 1 and that the weight for each input and output should be greater than or equal to 0. Mathematically, the function can be expressed through equation (2).

*Min* $\frac{\sum_{i} \nu_{i}x_{io}}{\sum_{r} u_{r}y_{ro}}$ (2)

subject to

$\frac{\sum_{i} \nu_{i}x_{ij}}{\sum_{r} u_{r}y_{rj}}\leq1$ for j=1,…, n,

*u_r_*, *v_i_* ≥ 0 for all *i* and *r*

where *u_r_* and *v_i_* are weights for the outputs (*y_rj_*) and inputs (*x_ij_*), respectively.

Without further restrictions, the objective function in this ratio form would have an infinite number of solutions. So we can arbitrarily set $\sum_{r} u_{r}y_{ro}=1$ without altering efficiency measurement. Thus, formula (2) becomes equation (3).

*Min* $\sum_{i} \nu_{i}x_{io}$ (3)

subject to

$$\sum_{r} u_{r}y_{rj}-\sum_{i} \nu_{i}x_{ij}\leq0$$

$\sum_{r} u_{r}y_{ro}=1$
*u_r_*, *v_i_* ≥ 0 for all *i* and *r*

This formulation turns out to be a linear programming problem. And its dual problem is given by equation (4).

*Max Φ*  (4)

subject to

$$\sum_{j=1}^{n} x_{ij}\lambda_{i}\leq x_{io}$$

$$\sum_{j=1}^{n} y_{ij}\lambda_{i}\geq\Phi y_{io}$$

*λ_j_*≥0

In formula (4), *Φ* is greater than or equal to 1, denoting the inverse of the efficiency score that measures technical efficiency of the *j*-th units. If *Φ* =1, then the DMU is at a frontier point in the graph. And *λ* with *Φ* >1 denotes an inefficient point within the boundary. Here *Φ* -1 is the proportion of increase in outputs that can be achieved by the DMU, holding the quantity of inputs constant. This is typically described as a DEA output-oriented model with constant returns to the scale (CRS), representing the percentage of increase of outputs that would push the DMUs to the efficient frontier. CRS means if all inputs to the entire system (i.e., global expenditures for HIV/AIDS) increased by a certain factor, then output would increase by the same factor. This CRS model assumes that all DMUs are operating at an optimal scale.

However, there are many reasons to cause a DMU not to be operating at its optimal scale. A model incorporating variable returns to scale (VRS) was developed to consider such situations simply by adding one more constraint of convexity ($\sum_{j=1}^{n} \lambda_{j}=1$) in the previous constant-returns-to-scale model. VRS DEA model is most widely applied in current research.

VRS output-oriented DEA can be illustrated graphically in a situation with one input and one output (Figure A1). DMUs A, B, C, D, and E are estimated to be efficient with *Φ* = 1, while DMU F with hypothetical input (HI) is an inefficient unit below the frontier, comparing with the virtual DMU of G, whose output is a linear combination of those in DMU C and DMU D. Here *Φ* equals *d_2_*/*d_1_*, the efficiency score is *d_1_*/*d_2_*, and *Φ* -1 is the proportion of increase of outputs needed to make the DMU to be on the frontier.

D

E

F

A

B

C

d_1_

d_2_

G

HI

Figure A1. Hypothetical output-oriented DEA model with variable returns to scale

An inefficient DMU can become efficient by increasing outputs while having the same amount of inputs, as mentioned above. It can also become efficient by reducing inputs while producing same amount of outputs. In this way, we can project the inefficient DMUs on horizontal axis, which is called the input-oriented DEA model. The mathematical expression of input-oriented DEA model is very similar to the output-oriented counterpart.

# Web-appendix 3. Boundary setting for outputs in DEA

One disadvantage of using conventional DEA (CCR model) is that the weights given to inputs and outputs are based on mathematical maximization or minimization, without incorporating prior knowledge of the relative importance of inputs and outputs. To overcome this limitation, we used the DEA model with an assurance region (AR), setting weight boundaries for outputs. The weight boundaries for outputs were based on the unit cost of each service in the analysis. We obtained the unit cost for PMTCT and VCT services from a manual for resource needs model (Table A1), and calculated the low quartile and upper quartile cost for PMTCT by adding the cost of each of the components for PMTCT, to derive the boundary of relative weights of PMTCT to VCT. The lower and upper boundaries were calculated as the ratio of costs of PMTCT in low quartile and upper quartile to those of VCT in low quartile, and rounded to 0.5 or 0.0. Therefore the boundary restriction of weight of PMTCT to VCT is$20>\frac{W_{(PMTCT)}}{W_{(VCT)}}>2.5$, where $W_{(PMTCT)}$is the weight for PMTCT services and $W_{(VCT)}$ is for VCT services.

The unit cost for ARV drugs was derived from UNAIDS financial report, which listed the unit costs for ARV in the countries of Ukraine and Mozambique. We used this information and derived the average cost with range (Table A2). Applying the assumption that 10% of AIDS patients needed second line drugs, we calculated the weighted unit cost for ARV for higher and lower cost scenarios. To estimate the unit cost for ARV therapy, we also included the costs for service delivery and laboratory monitoring. The information on service delivery was obtained from the methodological Annex III for estimating resource needs for AIDS in low- and middle-income countries. The highest and lowest regional average costs for service delivery were selected to use in the calculation. The laboratory costs were retrieved from the reviewed literature. Similarly, the highest and lowest costs available were used to calculate the unit costs for ARV treatment in two scenarios. The total costs on ARV drugs, laboratory tests and service delivery were used to calculate the relative prices of ARV to VCT as lower boundary and upper boundary, and the boundaries were rounded to 10. Considering most information on ARV treatment is from 2005, the cost of ARV treatment was reduced after local governments and international communities, including the Gates Foundation and Clinton Foundation, negotiated with pharmaceutical companies to reduce ARV drug prices. The boundary set is reasonable for the year 2006 and after. The restriction boundary is set as $180>\frac{W_{(ARV)}}{W_{(VCT)}}>40$

Table A1. Unit costs for PMTCT and VCT

| **Services** | **Average unit cost($)** | **Low quartile** | **Upper quartile** |
| --- | --- | --- | --- |
| PMTCT |  |  |  |
| Test | 8 | 4 | 10 |
| HIV+treatment | 40 | 10 | 165 |
| Formula | 24 | 14 | 47 |
| PMTCT total | 72 | 28 | 222 |
| VCT | 15 | 11 | 25 |

Source: Futures Group/Constella. Resource needs for HIV/AIDS: model for estimating resource needs for prevention, care, and mitigation. 2005

Table A2. Unit costs for ARV treatment

| **ARV** | **Higher unit cost ($)** | **Low unit cost ($)** |
| --- | --- | --- |
| Drugs |  |  |
| First line | 529 | 180 |
| Second Line | 3900 | 510 |
| Weighted drug costs | 866 | 213 |
| Laboratory monitoring | 759 | 128 |
| Service delivery | 345 | 72 |
| Total costs | 1970 | 413 |

Source: UNAIDS. Financial resources required to achieve universal access to HIV prevention, treatment, care and support. Geneva 2007; Bollinger L, Stover J. Financial resources required to achieve universal access to HIV prevention, treatment, care and support: Methodology for care and treatment interventions. Geneva 2007; Bautista SA, Dmytraczenko T, Kombe G, Bertozzi SM. Costing of HIV/AIDS Treatment in Mexico. PHRPlus report, June 2003; Cleary SM, McIntyre D, Boulle AM. The cost-effectiveness of Antiretroviral Treatment in Khayelitsha, South Africa - a primary data analysis. Cost Eff Resource Alloc, 2006

# Web-appendix 4. Supplemental table

Table A3. Data on spending and services for the countries in the analysis

| **Country** | **Year** | **Spending (Millions, current US$)** | **Spending (Millions, 2007 I$)** | **VCT Clients (Thousand)** | **Adult population (million)** | **ARV patients (Thousand)** | **ARV treatment needs (Thousand)** | **PMTCT (Thousand)** | **PPP converter** | **Exchange rate** |
| --- | --- | --- | --- | --- | --- | --- | --- | --- | --- | --- |
| Angola | 2006 | 27.72 | 46.37 | 280.78 | 5.62 | 6.51 | 40.71 | 1.92 | 49.42 | 80.18 |
| Angola | 2007 | 47.49 | 70.84 | 830.87 | 5.76 | 11.54 | 47.00 | 1.65 | 51.55 | 76.89 |
| Argentina | 2002 | 152.26 | 568.80 | 19.94 | 18.83 | 17.62 | 25.39 | 0.58 | 1.04 | 3.38 |
| Argentina | 2006 | 149.53 | 340.46 | 47.27 | 19.62 | 35.21 | 49.59 | 2.12 | 1.39 | 3.07 |
| Belarus | 2007 | 25.74 | 60.57 | 1610.62 | 5.20 | 0.88 | 4.30 | 0.13 | 912.58 | 2147.75 |
| Belize | 2006 | 5.08 | 9.98 | 21.83 | 0.15 | 0.44 | 1.04 | 0.06 | 1.05 | 2.00 |
| Benin | 2005 | 25.87 | 66.16 | 149.22 | 3.55 | 4.67 | 16.69 | 1.21 | 219.59 | 527.85 |
| Benin | 2006 | 25.93 | 64.23 | 514.52 | 3.68 | 7.63 | 18.18 | 2.38 | 219.28 | 526.82 |
| Benin | 2007 | 25.99 | 56.55 | 1141.74 | 3.80 | 9.77 | 20.00 | 1.83 | 219.08 | 476.66 |
| Botswana | 2003 | 150.32 | 384.51 | 49.89 | 0.88 | 10.00 | 66.00 | 5.76 | 2.17 | 4.94 |
| Botswana | 2004 | 207.98 | 469.33 | 122.96 | 0.90 | 37.00 | 84.09 | 10.51 | 2.24 | 4.60 |
| Botswana | 2005 | 206.94 | 459.40 | 271.47 | 0.92 | 59.95 | 93.67 | 7.54 | 2.42 | 5.05 |
| Botswana | 2006 | 143.41 | 314.23 | 496.34 | 0.95 | 79.49 | 104.59 | 12.99 | 2.71 | 5.76 |
| Botswana | 2007 | 229.46 | 466.69 | 723.61 | 0.97 | 92.93 | 120.00 | 12.42 | 2.95 | 6.00 |
| Brazil | 2005 | 574.78 | 1106.03 | 2437.43 | 104.81 | 174.00 | 217.50 | 6.77 | 1.36 | 2.46 |
| Brazil | 2006 | 565.19 | 920.44 | 7156.22 | 105.75 | 174.27 | 223.42 | 6.51 | 1.38 | 2.18 |
| Burkina Faso | 2003 | 24.26 | 82.51 | 34.00 | 5.87 | 1.50 | 59.00 | 0.04 | 191.90 | 580.88 |
| Burkina Faso | 2005 | 48.23 | 135.25 | 139.77 | 6.29 | 8.21 | 43.23 | 0.94 | 200.23 | 527.85 |
| Burkina Faso | 2006 | 43.28 | 121.27 | 511.09 | 6.51 | 14.08 | 45.42 | 1.62 | 193.81 | 526.82 |
| Burkina Faso | 2007 | 61.02 | 149.07 | 1416.10 | 6.74 | 16.94 | 48.00 | 1.48 | 195.10 | 476.66 |
| Cambodia | 2002 | 8.96 | 33.12 | 31.23 | 6.49 | 0.73 | 29.68 | 0.04 | 1217.37 | 3914.00 |
| Cambodia | 2004 | 19.68 | 69.80 | 86.14 | 6.95 | 5.00 | 35.71 | 0.16 | 1245.61 | 4020.78 |
| Cambodia | 2005 | 27.19 | 92.80 | 115.18 | 7.19 | 12.40 | 36.46 | 0.23 | 1278.54 | 4101.86 |
| Cambodia | 2006 | 44.18 | 143.97 | 297.29 | 7.43 | 20.13 | 37.28 | 0.31 | 1296.03 | 4096.82 |
| Cameroon | 2004 | 43.52 | 102.20 | 54.06 | 8.06 | 14.00 | 155.56 | 4.20 | 247.06 | 527.98 |
| Cameroon | 2005 | 43.17 | 96.58 | 116.04 | 8.29 | 23.82 | 170.14 | 3.59 | 251.02 | 527.86 |
| Cameroon | 2006 | 33.94 | 72.91 | 247.10 | 8.51 | 28.40 | 177.52 | 7.59 | 252.77 | 526.71 |
| Cameroon | 2007 | 37.83 | 71.79 | 517.38 | 8.74 | 45.82 | 180.00 | 7.52 | 251.14 | 476.66 |
| Central African Republic | 2006 | 14.69 | 29.95 | 327.69 | 2.05 | 2.78 | 46.37 | 1.94 | 266.43 | 526.71 |
| Chad | 2004 | 0.20 | 0.66 | 8.41 | 3.90 | 0.30 | 30.00 | 0.04 | 174.83 | 527.98 |
| Chad | 2006 | 1.73 | 4.40 | 61.59 | 4.16 | 5.50 | 50.00 | 0.25 | 213.94 | 526.71 |
| Chile | 2007 | 102.00 | 165.43 | 2420.61 | 8.65 | 10.22 | 12.00 | 0.12 | 370.96 | 601.63 |
| China | 2003 | 89.29 | 254.05 | 175.00 | 731.89 | 7.40 | 88.00 | 0.10 | 3.27 | 8.28 |
| China | 2005 | 119.89 | 302.79 | 400.00 | 744.02 | 19.28 | 148.32 | 0.14 | 3.45 | 8.19 |
| China | 2006 | 138.93 | 329.91 | 14900.00 | 747.46 | 31.14 | 163.90 | 0.65 | 3.46 | 7.97 |
| Colombia | 2006 | 97.65 | 216.66 | 544.91 | 23.52 | 17.54 | 51.59 | 0.11 | 1104.42 | 2377.00 |
| Congo, Dem. Rep. | 2006 | 41.03 | 89.17 | 473.67 | 27.63 | 17.56 | 117.07 | 3.42 | 237.04 | 499.67 |
| Congo, Dem. Rep. | 2007 | 54.32 | 105.73 | 1145.62 | 28.64 | 28.93 | 120.00 | 3.44 | 282.76 | 550.40 |
| Congo, Rep. | 2005 | 9.22 | 19.26 | 48.53 | 1.62 | 2.97 | 26.97 | 1.09 | 268.76 | 527.86 |
| Congo, Rep. | 2007 | 16.15 | 27.82 | 243.50 | 1.72 | 4.96 | 29.00 | 0.24 | 276.77 | 476.66 |
| Cote d'Ivoire | 2004 | 6.32 | 12.86 | 11.35 | 8.85 | 5.00 | 166.67 | 1.77 | 285.02 | 528.03 |
| Cote d'Ivoire | 2005 | 15.72 | 30.71 | 273.24 | 9.11 | 18.53 | 185.33 | 2.54 | 287.49 | 527.85 |
| Cote d'Ivoire | 2006 | 39.13 | 73.05 | 238.98 | 9.36 | 36.35 | 191.31 | 2.77 | 290.91 | 526.82 |
| Dominican Republic | 2005 | 7.03 | 12.33 | 113.12 | 4.73 | 2.58 | 21.52 | 0.68 | 18.22 | 30.05 |
| Dominican Republic | 2007 | 13.74 | 23.61 | 976.73 | 4.88 | 8.20 | 22.00 | 0.80 | 19.15 | 32.91 |
| Ecuador | 2004 | 2.40 | 6.44 | 37.58 | 6.92 | 1.00 | 6.25 | 0.09 | 0.41 | 1.00 |
| Ecuador | 2005 | 2.95 | 7.47 | 78.51 | 7.02 | 1.57 | 6.81 | 0.22 | 0.42 | 1.00 |
| Ecuador | 2007 | 7.47 | 16.25 | 336.84 | 7.27 | 3.21 | 7.60 | 0.27 | 0.46 | 1.00 |
| El Salvador | 2003 | 30.73 | 75.05 | 96.39 | 3.22 | 0.43 | 4.00 | 0.24 | 0.46 | 1.00 |
| El Salvador | 2005 | 35.94 | 81.35 | 222.70 | 3.36 | 2.87 | 9.27 | 0.14 | 0.47 | 1.00 |
| El Salvador | 2006 | 33.13 | 71.15 | 333.07 | 3.43 | 4.71 | 10.24 | 0.19 | 0.48 | 1.00 |
| Eritrea | 2006 | 7.18 | 20.76 | 94.50 | 2.42 | 1.18 | 9.79 | 0.08 | 5.35 | 15.00 |
| Eritrea | 2007 | 7.79 | 20.90 | 150.11 | 2.50 | 1.30 | 10.00 | 0.17 | 5.59 | 14.99 |
| Gabon | 2007 | 9.69 | 16.83 | 81.53 | 0.68 | 6.37 | 15.00 | 0.49 | 274.53 | 476.66 |
| Gambia, The | 2004 | 7.30 | 31.67 | 14.41 | 0.71 | 0.15 | 1.67 | 0.09 | 7.47 | 29.50 |
| Gambia, The | 2005 | 9.66 | 37.97 | 73.20 | 0.73 | 0.15 | 1.88 | 0.09 | 7.56 | 27.93 |
| Gambia, The | 2007 | 16.93 | 53.05 | 510.87 | 0.78 | 0.43 | 2.30 | 0.13 | 7.65 | 23.97 |
| Ghana | 2004 | 22.95 | 67.55 | 665.00 | 10.84 | 2.00 | 66.67 | 0.20 | 3333.94 | 8929.70 |
| Guatemala | 2003 | 15.85 | 39.39 | 12.61 | 5.38 | 2.74 | 12.00 | 0.07 | 3.59 | 7.94 |
| Guatemala | 2005 | 19.14 | 39.79 | 20.23 | 5.67 | 5.63 | 17.60 | 0.11 | 3.91 | 7.64 |
| Guatemala | 2006 | 18.96 | 37.37 | 25.77 | 5.86 | 6.03 | 19.45 | 0.17 | 3.98 | 7.61 |
| Guyana | 2003 | 2.07 | 7.10 | 2.12 | 0.44 | 0.25 | 2.00 | 0.06 | 64.16 | 195.50 |
| Guyana | 2005 | 2.49 | 8.00 | 48.87 | 0.44 | 1.20 | 4.14 | 0.11 | 66.28 | 200.67 |
| Haiti | 2003 | 18.13 | 66.92 | 53.95 | 3.69 | 1.37 | 47.00 | 0.22 | 12.46 | 40.92 |
| Haiti | 2006 | 70.28 | 153.21 | 283.51 | 4.05 | 8.80 | 33.83 | 0.99 | 19.05 | 40.28 |
| Honduras | 2003 | 20.61 | 63.34 | 34.76 | 3.34 | 1.42 | 8.00 | 0.07 | 6.34 | 17.34 |
| Honduras | 2006 | 14.35 | 38.42 | 313.38 | 3.66 | 4.67 | 11.40 | 0.20 | 7.28 | 18.90 |
| Indonesia | 2004 | 40.03 | 111.31 | 7.67 | 124.47 | 3.00 | 15.79 | 0.02 | 3530.96 | 8934.90 |
| Indonesia | 2006 | 56.58 | 123.09 | 29.29 | 127.26 | 5.10 | 34.00 | 0.04 | 4348.35 | 9176.82 |
| Iran | 2006 | 32.78 | 105.14 | 19.01 | 40.01 | 0.53 | 17.50 | 0.02 | 2948.18 | 9172.64 |
| Jamaica | 2004 | 9.20 | 20.28 | 218.18 | 1.36 | 0.50 | 7.14 | 0.32 | 30.24 | 60.68 |
| Jamaica | 2005 | 11.31 | 23.07 | 391.53 | 1.39 | 1.46 | 7.28 | 0.30 | 32.19 | 61.73 |
| Jamaica | 2006 | 10.36 | 20.15 | 632.58 | 1.41 | 2.63 | 7.98 | 0.37 | 34.51 | 65.11 |
| Jamaica | 2007 | 14.75 | 27.02 | 899.47 | 1.44 | 3.64 | 8.50 | 0.29 | 37.62 | 68.92 |
| Kazakhstan | 2005 | 4.68 | 11.48 | 440.25 | 8.58 | 0.24 | 1.04 | 0.05 | 57.61 | 132.90 |
| Kazakhstan | 2007 | 17.96 | 28.73 | 606.73 | 8.67 | 0.44 | 1.90 | 0.13 | 76.32 | 122.10 |
| Kenya | 2002 | 103.90 | 369.82 | 110.00 | 15.98 | 3.00 | 353.56 | 3.40 | 25.35 | 78.50 |
| Kenya | 2005 | 241.03 | 655.71 | 350.00 | 17.32 | 65.77 | 469.81 | 19.40 | 29.52 | 75.49 |
| Lao PDR | 2004 | 4.99 | 20.39 | 12.95 | 2.93 | 0.10 | 0.24 | 0.00 | 2850.68 | 10590.45 |
| Lao PDR | 2006 | 4.68 | 15.73 | 12.03 | 3.11 | 0.48 | 0.51 | 0.02 | 3092.04 | 10092.33 |
| Lao PDR | 2007 | 5.82 | 17.91 | 11.57 | 3.19 | 0.70 | 0.69 | 0.02 | 3114.37 | 9576.67 |
| Latvia | 2004 | 1.50 | 3.17 | 64.75 | 1.19 | 0.20 | 0.81 | 0.06 | 0.28 | 0.54 |
| Latvia | 2006 | 5.75 | 10.18 | 26.16 | 1.18 | 0.30 | 1.67 | 0.04 | 0.32 | 0.55 |
| Lesotho | 2005 | 18.46 | 35.84 | 16.97 | 1.07 | 8.40 | 76.36 | 1.81 | 3.49 | 6.37 |
| Lesotho | 2006 | 24.44 | 48.81 | 43.97 | 1.08 | 17.67 | 80.31 | 2.01 | 3.52 | 6.82 |
| Madagascar | 2007 | 16.82 | 41.66 | 48.44 | 9.04 | 0.14 | 3.20 | 0.03 | 749.24 | 1855.20 |
| Malawi | 2004 | 42.68 | 144.99 | 184.00 | 5.68 | 13.18 | 263.66 | 2.72 | 35.22 | 108.89 |
| Malawi | 2005 | 56.49 | 165.84 | 259.71 | 5.82 | 29.09 | 264.43 | 5.08 | 39.46 | 108.89 |
| Mali | 2005 | 20.22 | 47.30 | 19.05 | 4.89 | 7.04 | 25.14 | 0.42 | 240.09 | 527.85 |
| Mali | 2006 | 26.77 | 59.45 | 251.37 | 5.03 | 11.51 | 27.40 | 0.66 | 244.57 | 526.82 |
| Mauritius | 2006 | 1.50 | 3.23 | 272.54 | 0.69 | 0.24 | 1.01 | 0.00 | 14.84 | 31.02 |
| Mexico | 2002 | 226.05 | 407.76 | 108.00 | 54.57 | 6.90 | 19.76 | 0.21 | 6.20 | 9.73 |
| Mexico | 2004 | 206.41 | 367.61 | 1392.29 | 56.27 | 29.00 | 64.44 | 0.12 | 6.96 | 11.28 |
| Moldova | 2007 | 8.19 | 18.03 | 194.08 | 2.43 | 0.46 | 0.80 | 0.07 | 5.44 | 11.98 |
| Morocco | 2005 | 5.39 | 10.42 | 11.36 | 17.91 | 0.88 | 4.00 | 0.04 | 4.88 | 8.87 |
| Morocco | 2007 | 8.83 | 14.81 | 10.71 | 18.60 | 1.65 | 5.30 | 0.04 | 4.85 | 8.13 |
| Mozambique | 2004 | 46.85 | 109.57 | 184.37 | 8.94 | 7.00 | 233.33 | 3.12 | 10116.32 | 21531.11 |
| Mozambique | 2005 | 58.25 | 120.59 | 369.03 | 9.13 | 19.85 | 283.63 | 8.49 | 10909.46 | 21231.73 |
| Mozambique | 2006 | 95.51 | 220.19 | 724.49 | 9.32 | 40.89 | 340.76 | 12.15 | 11551.89 | 25834.50 |
| Namibia | 2005 | 79.12 | 125.57 | 65.00 | 1.02 | 29.20 | 47.10 | 4.06 | 4.27 | 6.37 |
| Namibia | 2007 | 130.50 | 186.09 | 244.12 | 1.06 | 52.32 | 59.00 | 18.51 | 4.93 | 7.03 |
| Nepal | 2005 | 7.48 | 25.06 | 5.91 | 13.69 | 0.08 | 10.20 | 0.02 | 22.65 | 71.31 |
| Nepal | 2006 | 8.90 | 28.35 | 15.26 | 14.07 | 0.54 | 18.03 | 0.04 | 23.48 | 72.57 |
| Nicaragua | 2002 | 8.04 | 26.71 | 42.00 | 2.64 | 0.01 | 0.85 | 0.00 | 4.95 | 14.30 |
| Niger | 2006 | 21.63 | 52.78 | 110.73 | 5.54 | 1.17 | 14.60 | 0.21 | 222.59 | 526.82 |
| Nigeria | 2005 | 19.14 | 44.12 | 62.12 | 65.63 | 41.22 | 687.07 | 0.53 | 60.23 | 130.50 |
| Pakistan | 2007 | 5.08 | 14.42 | 193.24 | 84.65 | 0.55 | 20.00 | 0.01 | 21.22 | 60.28 |
| Panama | 2003 | 13.39 | 25.94 | 5.57 | 1.60 | 1.53 | 2.00 | 0.05 | 0.58 | 1.00 |
| Paraguay | 2006 | 1.02 | 2.78 | 459.34 | 3.16 | 1.03 | 4.10 | 0.07 | 2107.12 | 5584.67 |
| Paraguay | 2007 | 2.33 | 5.28 | 844.33 | 3.24 | 1.06 | 4.80 | 0.14 | 2211.34 | 5016.17 |
| Peru | 2005 | 21.88 | 51.54 | 781.48 | 14.97 | 6.41 | 18.31 | 0.19 | 1.49 | 3.30 |
| Peru | 2006 | 32.39 | 71.33 | 1561.69 | 15.24 | 8.42 | 20.06 | 0.56 | 1.54 | 3.29 |
| Peru | 2007 | 28.01 | 56.93 | 2968.72 | 15.52 | 10.86 | 23.00 | 0.50 | 1.53 | 3.11 |
| Romania | 2004 | 19.81 | 54.60 | 206.00 | 11.79 | 6.00 | 7.32 | 0.02 | 1.30 | 3.26 |
| Romania | 2006 | 76.09 | 144.07 | 1667.61 | 11.66 | 6.79 | 8.38 | 0.08 | 1.53 | 2.81 |
| Russian | 2004 | 40.87 | 117.36 | 900.00 | 78.59 | 3.00 | 75.00 | 5.60 | 11.01 | 28.77 |
| Rwanda | 2003 | 17.44 | 64.00 | 110.70 | 4.40 | 2.00 | 45.00 | 2.59 | 163.98 | 535.40 |
| Rwanda | 2005 | 50.04 | 158.96 | 519.12 | 4.66 | 19.29 | 66.51 | 5.78 | 186.18 | 556.00 |
| Rwanda | 2006 | 84.74 | 235.34 | 1036.89 | 4.79 | 34.64 | 66.61 | 7.77 | 204.03 | 549.63 |
| Senegal | 2003 | 18.36 | 47.17 | 3.18 | 5.35 | 1.60 | 7.00 | 0.01 | 253.98 | 580.88 |
| Senegal | 2005 | 16.52 | 36.85 | 30.82 | 5.68 | 4.20 | 7.78 | 0.06 | 251.67 | 527.85 |
| Senegal | 2006 | 11.94 | 25.71 | 95.07 | 5.85 | 5.50 | 9.65 | 0.21 | 252.10 | 526.82 |
| Senegal | 2007 | 17.82 | 32.83 | 286.79 | 6.01 | 6.70 | 12.00 | 0.26 | 258.69 | 476.66 |
| South Africa | 2002 | 33.30 | 111.43 | 212.29 | 24.37 | 1.27 | 760.00 | 18.86 | 3.61 | 10.51 |
| South Africa | 2005 | 283.78 | 496.92 | 903.00 | 25.63 | 206.72 | 1378.12 | 75.08 | 3.87 | 6.37 |
| South Africa | 2006 | 797.22 | 1382.06 | 1443.59 | 26.03 | 324.75 | 1546.45 | 111.36 | 4.02 | 6.76 |
| South Africa | 2007 | 860.84 | 1421.30 | 2279.47 | 26.43 | 458.95 | 1700.00 | 127.16 | 4.27 | 7.05 |
| Swaziland | 2003 | 21.17 | 56.46 | 10.40 | 0.55 | 2.65 | 35.00 | 0.24 | 3.16 | 7.50 |
| Swaziland | 2004 | 28.03 | 60.93 | 31.33 | 0.55 | 6.00 | 42.86 | 0.59 | 3.22 | 6.37 |
| Swaziland | 2006 | 49.11 | 98.94 | 61.10 | 0.56 | 18.49 | 52.84 | 8.22 | 3.49 | 6.82 |
| Swaziland | 2007 | 65.01 | 123.19 | 90.19 | 0.56 | 24.54 | 59.00 | 8.77 | 3.71 | 7.03 |
| Tanzania | 2005 | 266.37 | 810.87 | 1547.83 | 17.55 | 21.54 | 430.86 | 6.20 | 395.63 | 1132.09 |
| Tanzania | 2006 | 323.50 | 1030.26 | 3414.18 | 18.05 | 60.34 | 431.01 | 14.76 | 403.53 | 1246.58 |
| Tanzania | 2007 | 392.88 | 1131.28 | 6688.24 | 18.58 | 135.70 | 440.00 | 31.86 | 428.21 | 1233.00 |
| Thailand | 2004 | 122.90 | 346.24 | 5829.98 | 35.98 | 50.00 | 238.10 | 5.02 | 15.70 | 40.25 |
| Thailand | 2006 | 161.86 | 390.25 | 6875.24 | 36.19 | 112.20 | 243.90 | 4.31 | 16.21 | 37.91 |
| Thailand | 2007 | 199.64 | 394.63 | 7448.04 | 36.28 | 152.97 | 250.00 | 9.35 | 16.29 | 32.20 |
| Togo | 2004 | 4.32 | 10.21 | 13.48 | 2.53 | 2.00 | 33.33 | 0.18 | 245.34 | 528.03 |
| Togo | 2006 | 8.44 | 19.63 | 153.53 | 2.71 | 6.99 | 38.85 | 0.91 | 233.35 | 526.82 |
| Togo | 2007 | 11.79 | 24.52 | 446.69 | 2.79 | 7.98 | 41.00 | 0.71 | 229.27 | 476.66 |
| Uganda | 2005 | 202.42 | 614.36 | 1000.00 | 12.12 | 74.87 | 356.52 | 12.07 | 619.64 | 1767.83 |
| Uganda | 2006 | 267.96 | 819.92 | 1513.93 | 12.62 | 96.29 | 356.64 | 19.99 | 614.42 | 1823.63 |
| Ukraine | 2004 | 28.03 | 118.12 | 38.33 | 24.57 | 1.00 | 50.00 | 2.82 | 1.39 | 5.33 |
| Ukraine | 2005 | 39.41 | 126.54 | 10.94 | 24.45 | 3.45 | 69.00 | 2.17 | 1.68 | 5.07 |
| Uruguay | 2002 | 12.02 | 25.91 | 38.60 | 1.64 | 0.78 | 0.93 | 0.11 | 11.19 | 20.98 |
| Uruguay | 2006 | 5.73 | 10.30 | 229.24 | 1.69 | 1.50 | 2.72 | 0.05 | 13.72 | 23.93 |
| Vietnam | 2005 | 39.66 | 141.18 | 967.06 | 48.35 | 3.00 | 50.00 | 0.37 | 4712.75 | 15767.89 |
| Vietnam | 2006 | 47.16 | 158.44 | 1887.33 | 49.24 | 8.31 | 59.36 | 1.39 | 4897.41 | 15961.64 |
| Zambia | 2004 | 120.85 | 299.28 | 725.00 | 5.05 | 20.00 | 285.71 | 12.98 | 2090.05 | 4710.00 |
| Zambia | 2006 | 189.93 | 267.91 | 750.30 | 5.28 | 82.03 | 315.50 | 25.58 | 2661.77 | 3642.00 |
| Zambia | 2007 | 238.10 | 331.97 | 811.30 | 5.41 | 151.20 | 330.00 | 35.31 | 2839.08 | 3958.42 |
| Mean | | 73.67 | 166.03 | 734.12 | 28.01 | 24.84 | 115.11 | 5.11 | - | - |
| Standard error of the mean | | 10.48 | 21.06 | 140.99 | 8.46 | 4.60 | 19.66 | 1.27 | - | - |
| Median | | 25.99 | 66.16 | 212.29 | 5.76 | 6.03 | 35.00 | 0.42 | - | - |
| Standard deviation | | 129.18 | 259.71 | 1738.27 | 104.26 | 56.65 | 242.38 | 15.66 | - | - |
| Minimum | | 0.20 | 0.66 | 2.12 | 0.15 | 0.01 | 0.24 | 0.00 | - | - |
| Maximum | | 860.84 | 1421.30 | 14900.00 | 747.46 | 458.95 | 1700.00 | 127.16 | - | - |
| Sum | | 11124.12 | 25070.39 | 110851.73 | 4229.64 | 3751.30 | 17381.13 | 771.71 | - | - |

Notes: PPP converter: the ratio of national currency value of Gross Domestic Products (GDP) to the real value of GDP in international dollars; Exchange rate: the ratio at which the national currency is converted to US$

Table A4. Efficiency from various DEA models

| **Country** | **Region** | **Year** | **Efficiency scores** | | | **Saved spending (Million, I$ in 2007)** |  | **Projected volume of services (Thousand)** | | | | |
| --- | --- | --- | --- | --- | --- | --- | --- | --- | --- | --- | --- | --- |
|  |  |  | **Output-oriented by year** | **Input-oriented by year** | **Pooled output-oriented** |  |  | **PMTCT** | | **VCT** | | **ARV** |
| C2 | LA | 2002 | 100.00% | 100.00% | 7.34% | 0.00 |  | 0.58 | | 19.94 | | 17.62 |
| C9 | SSEA | 2002 | 74.65% | 78.25% | 3.45% | 7.20 |  | 0.39 | | 40.98 | | 0.97 |
| C34 | SSA | 2002 | 43.76% | 33.35% | 2.23% | 246.48 |  | 8.53 | | 103.62 | | 10.51 |
| C42 | LA | 2002 | 66.35% | 60.64% | 3.79% | 160.51 |  | 7.01 | | 87.67 | | 11.86 |
| C48 | LA | 2002 | 58.41% | 97.01% | 2.19% | 0.80 |  | 0.28 | | 40.22 | | 0.79 |
| C59 | SSA | 2002 | 100.00% | 100.00% | 10.75% | 0.00 |  | 18.86 | | 212.29 | | 1.27 |
| C66 | LA | 2002 | 100.00% | 100.00% | 4.73% | 0.00 |  | 0.11 | | 38.60 | | 0.78 |
| C6 | SSA | 2003 | 100.00% | 100.00% | 5.72% | 0.00 |  | 5.76 | | 49.89 | | 10.00 |
| C8 | SSA | 2003 | 46.12% | 37.68% | 2.98% | 51.41 |  | 0.91 | | 71.64 | | 3.25 |
| C14 | EA | 2003 | 100.00% | 100.00% | 6.29% | 0.00 |  | 0.10 | | 175.00 | | 7.40 |
| C21 | LA | 2003 | 53.62% | 49.55% | 2.85% | 37.86 |  | 2.44 | | 114.44 | | 2.31 |
| C26 | LA | 2003 | 100.00% | 100.00% | 7.59% | 0.00 |  | 0.07 | | 12.61 | | 2.74 |
| C27 | LA | 2003 | 100.00% | 100.00% | 3.26% | 0.00 |  | 0.06 | | 2.12 | | 0.25 |
| C28 | CB | 2003 | 56.81% | 53.38% | 3.76% | 31.19 |  | 1.90 | | 91.19 | | 2.41 |
| C29 | LA | 2003 | 52.17% | 48.03% | 3.48% | 32.91 |  | 1.13 | | 64.16 | | 2.72 |
| C52 | LA | 2003 | 89.23% | 90.79% | 6.21% | 2.39 |  | 0.06 | | 8.24 | | 1.70 |
| C57 | SSA | 2003 | 100.00% | 100.00% | 7.54% | 0.00 |  | 2.59 | | 110.70 | | 2.00 |
| C58 | SSA | 2003 | 53.70% | 51.45% | 3.77% | 22.90 |  | 0.07 | | 18.50 | | 2.91 |
| C60 | SSA | 2003 | 83.87% | 68.34% | 5.81% | 17.87 |  | 0.07 | | 25.53 | | 3.11 |
| C6 | SSA | 2004 | 46.84% | 33.45% | 17.94% | 312.32 |  | 5.02 | | 5829.98 | | 50.00 |
| C9 | SSEA | 2004 | 29.95% | 22.24% | 10.65% | 54.27 |  | 2.33 | | 1005.09 | | 12.69 |
| C10 | SSA | 2004 | 56.63% | 52.51% | 22.01% | 48.54 |  | 2.64 | | 1570.57 | | 17.06 |
| C12 | SSA | 2004 | 100.00% | 100.00% | 100.00% | 0.00 |  | 0.03 | | 8.41 | | 0.30 |
| C18 | SSA | 2004 | 100.00% | 100.00% | 39.17% | 0.00 |  | 1.77 | | 11.35 | | 5.00 |
| C20 | LA | 2004 | 57.73% | 54.98% | 16.24% | 2.90 |  | 0.44 | | 64.37 | | 1.73 |
| C24 | SSA | 2004 | 3.05% | 2.24% | 1.14% | 30.96 |  | 0.57 | | 519.77 | | 4.92 |
| C25 | SSA | 2004 | 48.58% | 48.39% | 19.16% | 34.86 |  | 1.00 | | 1135.26 | | 9.92 |
| C30 | SSEA | 2004 | 10.89% | 6.83% | 4.29% | 103.71 |  | 2.73 | | 1729.55 | | 18.29 |
| C32 | CB | 2004 | 52.03% | 51.41% | 15.08% | 9.85 |  | 0.32 | | 338.95 | | 3.12 |
| C35 | SSEA | 2004 | 3.67% | 3.25% | 1.17% | 19.73 |  | 0.32 | | 340.74 | | 3.14 |
| C36 | EECA | 2004 | 94.39% | 93.96% | 14.21% | 0.19 |  | 0.07 | | 50.70 | | 0.66 |
| C39 | SSA | 2004 | 40.24% | 36.24% | 16.89% | 92.44 |  | 3.06 | | 2317.42 | | 22.83 |
| C42 | LA | 2004 | 44.55% | 40.60% | 20.12% | 218.38 |  | 5.02 | | 5829.98 | | 50.00 |
| C45 | SSA | 2004 | 30.12% | 23.92% | 12.44% | 83.36 |  | 2.71 | | 1699.21 | | 18.05 |
| C55 | EECA | 2004 | 48.36% | 41.31% | 17.87% | 32.04 |  | 2.18 | | 739.86 | | 10.63 |
| C56 | EECA | 2004 | 42.05% | 41.92% | 18.27% | 68.16 |  | 1.72 | | 1974.28 | | 17.08 |
| C60 | SSA | 2004 | 38.06% | 28.85% | 12.73% | 43.35 |  | 2.24 | | 850.25 | | 11.49 |
| C62 | SSEA | 2004 | 100.00% | 100.00% | 55.58% | 0.00 |  | 5.02 | | 5829.98 | | 50.00 |
| C63 | SSA | 2004 | 52.70% | 50.75% | 18.92% | 5.03 |  | 1.30 | | 23.19 | | 3.80 |
| C65 | EECA | 2004 | 5.12% | 4.76% | 2.50% | 112.50 |  | 2.80 | | 1848.50 | | 19.21 |
| C68 | SSA | 2004 | 35.37% | 33.28% | 16.78% | 199.69 |  | 4.56 | | 5010.43 | | 43.66 |
| C5 | SSA | 2005 | 16.71% | 17.65% | 12.33% | 54.48 |  | 4.16 | | 103.04 | | 49.28 |
| C6 | SSA | 2005 | 31.00% | 22.18% | 29.15% | 357.50 |  | 68.90 | | 833.33 | | 193.01 |
| C7 | LA | 2005 | 100.00% | 100.00% | 49.38% | 0.00 |  | 6.77 | | 2437.43 | | 174.00 |
| C8 | SSA | 2005 | 14.48% | 10.90% | 11.14% | 120.50 |  | 15.53 | | 231.35 | | 74.53 |
| C9 | SSEA | 2005 | 24.13% | 19.48% | 20.76% | 74.72 |  | 8.55 | | 152.52 | | 59.02 |
| C10 | SSA | 2005 | 41.48% | 30.70% | 38.46% | 66.93 |  | 9.17 | | 159.55 | | 60.40 |
| C14 | EA | 2005 | 19.27% | 9.95% | 13.93% | 272.65 |  | 43.12 | | 542.48 | | 135.76 |
| C17 | SSA | 2005 | 26.17% | 45.21% | 17.80% | 10.55 |  | 0.21 | | 264.73 | | 11.34 |
| C18 | SSA | 2005 | 88.81% | 88.78% | 71.78% | 3.45 |  | 0.33 | | 217.45 | | 24.39 |
| C19 | CB | 2005 | 56.48% | 73.46% | 26.22% | 3.27 |  | 0.18 | | 220.59 | | 4.57 |
| C20 | LA | 2005 | 100.00% | 100.00% | 24.36% | 0.00 |  | 0.22 | | 78.51 | | 1.57 |
| C21 | LA | 2005 | 14.44% | 13.25% | 8.82% | 70.57 |  | 6.66 | | 131.25 | | 54.83 |
| C24 | SSA | 2005 | 5.46% | 19.68% | 3.25% | 30.50 |  | 0.44 | | 133.32 | | 33.51 |
| C26 | LA | 2005 | 15.95% | 27.49% | 15.29% | 28.85 |  | 0.47 | | 112.27 | | 35.79 |
| C27 | LA | 2005 | 58.36% | 93.39% | 16.78% | 0.53 |  | 0.22 | | 83.65 | | 2.06 |
| C32 | CB | 2005 | 50.53% | 51.89% | 26.44% | 11.10 |  | 0.22 | | 306.02 | | 14.79 |
| C33 | EECA | 2005 | 100.00% | 100.00% | 37.84% | 0.00 |  | 0.05 | | 440.25 | | 0.24 |
| C34 | SSA | 2005 | 32.35% | 19.44% | 25.95% | 528.27 |  | 68.00 | | 1061.93 | | 203.33 |
| C37 | SSA | 2005 | 27.81% | 38.07% | 25.56% | 22.20 |  | 0.41 | | 158.00 | | 30.83 |
| C39 | SSA | 2005 | 38.11% | 23.55% | 32.72% | 126.78 |  | 20.57 | | 288.16 | | 85.71 |
| C40 | SSA | 2005 | 17.31% | 25.90% | 16.76% | 35.05 |  | 1.06 | | 68.03 | | 42.39 |
| C44 | MENA | 2005 | 18.19% | 71.72% | 8.46% | 2.95 |  | 0.25 | | 77.19 | | 4.76 |
| C45 | SSA | 2005 | 41.22% | 28.31% | 30.50% | 86.46 |  | 13.12 | | 204.13 | | 69.17 |
| C46 | SSA | 2005 | 40.92% | 27.11% | 38.80% | 91.53 |  | 13.94 | | 213.37 | | 70.99 |
| C47 | SSEA | 2005 | 0.96% | 29.82% | 0.59% | 17.59 |  | 0.25 | | 282.86 | | 17.30 |
| C50 | SSA | 2005 | 100.00% | 100.00% | 100.00% | 0.00 |  | 0.53 | | 62.12 | | 41.22 |
| C54 | LA | 2005 | 56.50% | 51.79% | 36.03% | 24.85 |  | 1.75 | | 75.90 | | 43.94 |
| C57 | SSA | 2005 | 35.74% | 22.66% | 24.81% | 122.94 |  | 19.44 | | 275.39 | | 83.20 |
| C58 | SSA | 2005 | 13.90% | 26.23% | 12.64% | 27.19 |  | 0.42 | | 146.31 | | 32.10 |
| C59 | SSA | 2005 | 100.00% | 100.00% | 98.11% | 0.00 |  | 75.08 | | 903.00 | | 206.72 |
| C61 | SSA | 2005 | 25.83% | 10.65% | 13.28% | 724.55 |  | 39.87 | | 1693.88 | | 189.85 |
| C64 | SSA | 2005 | 42.96% | 29.47% | 31.75% | 433.31 |  | 61.91 | | 1198.86 | | 200.41 |
| C65 | EECA | 2005 | 5.74% | 7.17% | 4.82% | 117.47 |  | 14.10 | | 215.17 | | 71.35 |
| C67 | SSEA | 2005 | 32.46% | 19.84% | 15.09% | 113.17 |  | 16.51 | | 242.36 | | 76.70 |
| C1 | SSA | 2006 | 35.59% | 24.03% | 24.85% | 35.23 |  | 1.21 | | 872.74 | | 18.30 |
| C2 | LA | 2006 | 26.82% | 20.06% | 20.81% | 272.17 |  | 4.06 | | 5833.59 | | 100.29 |
| C4 | LA | 2006 | 7.27% | 27.86% | 5.37% | 7.20 |  | 0.27 | | 310.76 | | 5.99 |
| C5 | SSA | 2006 | 36.19% | 27.92% | 27.31% | 46.29 |  | 1.37 | | 1525.86 | | 21.10 |
| C6 | SSA | 2006 | 67.59% | 64.12% | 52.27% | 112.74 |  | 3.94 | | 5284.75 | | 94.02 |
| C7 | LA | 2006 | 92.00% | 84.12% | 75.44% | 146.21 |  | 60.33 | | 7645.57 | | 189.43 |
| C8 | SSA | 2006 | 31.41% | 25.61% | 23.72% | 90.21 |  | 2.85 | | 1673.06 | | 44.83 |
| C9 | SSEA | 2006 | 34.63% | 27.56% | 25.59% | 104.29 |  | 3.12 | | 1722.68 | | 53.31 |
| C10 | SSA | 2006 | 82.09% | 80.05% | 56.51% | 14.55 |  | 2.61 | | 433.75 | | 34.60 |
| C11 | SSA | 2006 | 32.74% | 15.14% | 24.91% | 25.41 |  | 0.38 | | 1111.96 | | 8.50 |
| C12 | SSA | 2006 | 100.00% | 100.00% | 100.00% | 0.00 |  | 0.25 | | 61.59 | | 5.50 |
| C14 | EA | 2006 | 100.00% | 100.00% | 100.00% | 0.00 |  | 0.65 | | 14900.00 | | 31.14 |
| C15 | LA | 2006 | 23.18% | 17.87% | 17.60% | 177.95 |  | 3.47 | | 3243.37 | | 70.69 |
| C16 | SSA | 2006 | 47.97% | 42.97% | 35.24% | 50.85 |  | 2.67 | | 1076.78 | | 36.61 |
| C18 | SSA | 2006 | 100.00% | 100.00% | 69.92% | 0.00 |  | 2.77 | | 238.98 | | 36.35 |
| C21 | LA | 2006 | 20.21% | 11.01% | 15.35% | 63.32 |  | 1.54 | | 1646.86 | | 23.32 |
| C22 | SSA | 2006 | 14.49% | 13.40% | 10.20% | 17.98 |  | 0.40 | | 654.44 | | 8.11 |
| C26 | LA | 2006 | 29.20% | 13.67% | 17.47% | 32.26 |  | 1.46 | | 146.78 | | 20.31 |
| C28 | CB | 2006 | 15.76% | 10.20% | 11.83% | 137.58 |  | 3.16 | | 1915.91 | | 55.51 |
| C29 | LA | 2006 | 33.40% | 19.25% | 23.88% | 31.02 |  | 0.85 | | 937.61 | | 13.99 |
| C30 | SSEA | 2006 | 9.48% | 3.24% | 6.92% | 119.11 |  | 3.01 | | 1285.89 | | 48.31 |
| C31 | SSEA | 2006 | 1.31% | 2.65% | 0.98% | 102.35 |  | 2.73 | | 1450.53 | | 40.14 |
| C32 | CB | 2006 | 61.12% | 46.11% | 47.69% | 10.86 |  | 0.17 | | 1043.72 | | 4.31 |
| C35 | SSEA | 2006 | 5.12% | 17.68% | 3.48% | 12.95 |  | 0.55 | | 234.73 | | 9.35 |
| C36 | EECA | 2006 | 6.10% | 27.31% | 4.31% | 7.40 |  | 0.22 | | 436.41 | | 4.93 |
| C37 | SSA | 2006 | 68.06% | 62.73% | 41.67% | 18.19 |  | 1.88 | | 176.34 | | 25.45 |
| C40 | SSA | 2006 | 42.73% | 34.65% | 28.76% | 38.85 |  | 1.96 | | 587.25 | | 26.93 |
| C41 | SSA | 2006 | 54.05% | 85.99% | 49.46% | 0.45 |  | 0.07 | | 479.34 | | 1.07 |
| C45 | SSA | 2006 | 51.21% | 44.10% | 38.92% | 123.08 |  | 3.48 | | 3317.33 | | 71.53 |
| C47 | SSEA | 2006 | 3.88% | 9.81% | 2.52% | 25.57 |  | 0.91 | | 393.05 | | 13.94 |
| C49 | SSA | 2006 | 7.44% | 5.27% | 5.64% | 50.00 |  | 0.92 | | 1526.63 | | 15.69 |
| C53 | LA | 2006 | 100.00% | 100.00% | 100.00% | 0.00 |  | 0.07 | | 459.34 | | 1.03 |
| C54 | LA | 2006 | 58.28% | 53.41% | 48.96% | 33.23 |  | 0.69 | | 2685.25 | | 14.46 |
| C55 | EECA | 2006 | 29.42% | 25.44% | 26.95% | 107.42 |  | 1.05 | | 5665.33 | | 23.08 |
| C57 | SSA | 2006 | 43.10% | 36.51% | 32.87% | 149.43 |  | 3.56 | | 3634.33 | | 75.15 |
| C58 | SSA | 2006 | 39.27% | 19.51% | 24.53% | 20.70 |  | 0.93 | | 241.09 | | 14.00 |
| C59 | SSA | 2006 | 100.00% | 100.00% | 72.86% | 0.00 |  | 111.36 | | 1443.59 | | 324.75 |
| C60 | SSA | 2006 | 41.84% | 34.84% | 30.06% | 64.47 |  | 2.90 | | 780.62 | | 42.54 |
| C61 | SSA | 2006 | 37.79% | 19.17% | 30.49% | 832.73 |  | 49.11 | | 9009.26 | | 159.67 |
| C62 | SSEA | 2006 | 100.00% | 100.00% | 81.43% | 0.00 |  | 4.31 | | 6875.24 | | 112.20 |
| C63 | SSA | 2006 | 63.55% | 48.10% | 41.42% | 10.19 |  | 0.68 | | 256.53 | | 11.00 |
| C64 | SSA | 2006 | 45.62% | 32.54% | 33.87% | 553.14 |  | 50.68 | | 4522.17 | | 204.28 |
| C66 | LA | 2006 | 40.02% | 26.99% | 28.21% | 7.52 |  | 0.17 | | 572.69 | | 3.74 |
| C67 | SSEA | 2006 | 31.45% | 27.87% | 28.85% | 114.28 |  | 1.28 | | 6063.68 | | 26.42 |
| C68 | SSA | 2006 | 82.96% | 80.82% | 63.69% | 51.39 |  | 3.71 | | 4315.69 | | 82.94 |
| C1 | SSA | 2007 | 40.46% | 30.93% | 38.60% | 48.93 |  | 3.59 | | 2063.28 | | 28.53 |
| C3 | EECA | 2007 | 46.85% | 34.44% | 46.06% | 39.71 |  | 0.60 | | 3016.95 | | 12.39 |
| C5 | SSA | 2007 | 52.40% | 43.27% | 50.61% | 32.08 |  | 2.19 | | 2205.14 | | 18.64 |
| C6 | SSA | 2007 | 45.57% | 38.53% | 45.57% | 286.88 |  | 17.62 | | 7085.26 | | 174.45 |
| C8 | SSA | 2007 | 34.07% | 27.14% | 33.16% | 108.62 |  | 2.93 | | 4184.09 | | 49.71 |
| C10 | SSA | 2007 | 100.00% | 100.00% | 92.65% | 0.00 |  | 7.52 | | 517.38 | | 45.82 |
| C13 | LA | 2007 | 42.38% | 28.18% | 34.91% | 118.80 |  | 3.35 | | 4407.82 | | 56.52 |
| C16 | SSA | 2007 | 58.57% | 52.18% | 55.80% | 50.56 |  | 5.93 | | 1955.93 | | 49.39 |
| C17 | SSA | 2007 | 31.34% | 22.10% | 28.08% | 21.67 |  | 2.55 | | 772.52 | | 15.81 |
| C19 | CB | 2007 | 86.54% | 80.44% | 82.13% | 4.62 |  | 1.33 | | 1127.58 | | 9.47 |
| C20 | LA | 2007 | 40.30% | 32.48% | 37.66% | 10.97 |  | 1.26 | | 834.42 | | 7.98 |
| C22 | SSA | 2007 | 14.66% | 25.25% | 13.89% | 15.62 |  | 1.29 | | 1023.95 | | 8.88 |
| C23 | SSA | 2007 | 51.67% | 41.46% | 40.77% | 9.85 |  | 1.42 | | 787.56 | | 8.83 |
| C24 | SSA | 2007 | 16.47% | 9.95% | 16.47% | 47.77 |  | 0.47 | | 2809.12 | | 10.12 |
| C32 | CB | 2007 | 55.81% | 38.31% | 55.14% | 16.67 |  | 0.58 | | 1611.49 | | 6.52 |
| C33 | EECA | 2007 | 30.80% | 18.36% | 30.80% | 23.46 |  | 0.30 | | 1808.97 | | 5.51 |
| C35 | SSEA | 2007 | 5.49% | 29.46% | 4.31% | 12.63 |  | 1.54 | | 782.24 | | 9.56 |
| C38 | SSA | 2007 | 2.04% | 12.67% | 2.04% | 36.38 |  | 0.40 | | 2340.49 | | 7.96 |
| C43 | EECA | 2007 | 14.16% | 29.27% | 14.16% | 12.75 |  | 0.23 | | 1368.74 | | 3.48 |
| C44 | MENA | 2007 | 14.33% | 35.63% | 11.26% | 9.53 |  | 1.20 | | 797.49 | | 7.47 |
| C46 | SSA | 2007 | 55.09% | 45.96% | 53.63% | 100.57 |  | 8.17 | | 2971.05 | | 83.75 |
| C51 | SSEA | 2007 | 16.52% | 36.58% | 16.38% | 9.15 |  | 0.32 | | 1169.21 | | 3.33 |
| C53 | LA | 2007 | 100.00% | 100.00% | 100.00% | 0.00 |  | 0.14 | | 844.33 | | 1.06 |
| C54 | LA | 2007 | 100.00% | 100.00% | 100.00% | 0.00 |  | 0.50 | | 2968.72 | | 10.86 |
| C58 | SSA | 2007 | 35.83% | 28.06% | 31.88% | 23.61 |  | 3.00 | | 794.68 | | 18.69 |
| C59 | SSA | 2007 | 100.00% | 100.00% | 100.00% | 0.00 |  | 127.16 | | 2279.47 | | 458.95 |
| C60 | SSA | 2007 | 35.75% | 29.26% | 34.08% | 87.14 |  | 7.81 | | 1620.86 | | 62.88 |
| C61 | SSA | 2007 | 64.61% | 31.99% | 61.98% | 769.33 |  | 93.88 | | 3739.50 | | 372.52 |
| C62 | SSEA | 2007 | 100.00% | 100.00% | 100.00% | 0.00 |  | 9.35 | | 7448.04 | | 152.97 |
| C63 | SSA | 2007 | 57.85% | 51.28% | 52.42% | 11.94 |  | 2.23 | | 769.67 | | 13.80 |
| C68 | SSA | 2007 | 95.54% | 95.03% | 95.07% | 16.49 |  | 9.00 | | 6102.91 | | 132.18 |
| Analysis of all DMUs | |  | | | | | | | | | | |
| Mean |  |  | 49.75% | 47.77% | 30.32% | 75.55 |  | 8.43 | | 1553.19 | | 45.92 |
| Standard error | |  | 2.53% | 2.59% | 2.22% | 11.08 |  | 1.64 | | 178.38 | | 5.69 |
| Median |  |  | 43.10% | 36.51% | 23.88% | 30.50 |  | 1.77 | | 780.62 | | 18.29 |
| Std |  |  | 31.19% | 31.98% | 27.31% | 136.62 |  | 20.23 | | 2199.19 | | 70.09 |
| Minimum |  |  | 0.96% | 2.24% | 0.59% | 0.00 |  | 0.03 | | 2.12 | | 0.24 |
| Maximum |  |  | 100.00% | 100.00% | 100.00% | 832.73 |  | 127.16 | | 14900.00 | | 458.95 |
| Sum |  |  | - | - | - | 11408.07 |  | 1272.87 | | 234531.53 | | 6934.06 |
| Expected increase (%) | | | |  |  |  |  | 164.94% | 211.57% | | 184.84% | |
| Expected increase (Thousands) | | | |  |  |  |  | 501.16 | 123679.80 | | 3182.76 | |
| Analysis of inefficient DMUs | | | | | | | | | | | | |
| Mean |  |  | 38.59% | 35.35% | 25.00% | 92.00 |  | 7.19 | | 1500.83 | | 42.20 |
| Standard error | |  | 2.10% | 2.03% | 1.79% | 13.09 |  | 1.42 | | 167.38 | | 5.12 |
| Median |  |  | 35.67% | 29.41% | 19.81% | 38.36 |  | 1.89 | | 796.09 | | 18.67 |
| Std |  |  | 23.69% | 22.90% | 20.19% | 145.73 |  | 15.84 | | 1863.84 | | 57.04 |
| Minimum |  |  | 1.06% | 2.26% | 0.59% | 0.19 |  | 0.06 | | 8.24 | | 0.66 |
| Maximum |  |  | 97.60% | 97.01% | 91.62% | 832.73 |  | 93.88 | | 9009.26 | | 372.52 |
| Sum |  |  | - | - | - | 11408.07 |  | 891.19 | | 186102.65 | | 5232.32 |
| Expected increase (%) | | | |  |  |  |  | 228.50% | 298.13% | | 255.29% | |
| Expected increase (Thousands) | | | |  |  |  |  | 501.16 | 123679.80 | | 3182.76 | |

Note: to preserve countries’ anonymity, the results did not identify countries by name. The input-oriented efficiency score shows the ratio of the minimal spending to the current spending to provide the same level of services.

SSA: Sub-Saharan Africa; SSEA: South & South East Asia; EA: East Asia; LA: Latin America; EECA: Eastern Europe & Central Asia; CB: Caribbean; MENA: Middle East & North Africa

Table A5. Determinants of the efficiency of national HIV/AIDS programs: Full specification

| **Determinant (independent variable)** | **Coefficient** | **Standard Error** | **t value** | **P value** |
| --- | --- | --- | --- | --- |
| Voice and accountability (VA)** | 0.392 | 0.150 | 2.613 | 0.009 |
| Government effectiveness (GE) | 0.500 | 0.353 | 1.416 | 0.157 |
| Rule of law (RL) | -0.333 | 0.344 | -0.968 | 0.333 |
| Control of corruption (CC) | -0.323 | 0.310 | -1.042 | 0.297 |
| Government commitment to health* | 0.031 | 0.015 | 2.067 | 0.042 |
| External source as percentage of total health expenditure ** | 0.030 | 0.010 | 3.000 | 0.004 |
| Government source as percentage of total health expenditure * | -0.014 | 0.006 | -2.333 | 0.023 |
| CC ×RL ** | 0.602 | 0.226 | 2.664 | 0.008 |
| CC × external share of health spending* | 0.022 | 0.009 | 2.444 | 0.013 |
| Log(GNI per capita)* | 3.485 | 1.442 | 2.417 | 0.016 |
| Square of log(GNI per capita)* | -0.207 | 0.093 | -2.226 | 0.026 |
| Log (adult population)* | 0.147 | 0.066 | 2.227 | 0.026 |
| HIV/AIDS prevalence (in percentage points)** | 0.052 | 0.015 | 3.467 | 0.000 |
| Reference category: year 2002 |  |  |  |  |
| Year 2003** | 0.485 | 0.160 | 3.031 | 0.002 |
| Year 2004** | 1.323 | 0.143 | 9.252 | 0.000 |
| Year 2005** | 1.702 | 0.143 | 11.902 | 0.000 |
| Year 2006** | 2.108 | 0.142 | 14.845 | 0.000 |
| Constant | -20.497 | 5.829 | -3.516 | 0.000 |

Source: Authors’ analysis of determinants of the efficiency of national HIV/AIDS programs, based on the efficiency scores generated from the DEA model. Notes: ** p<0.01, * p< 0.05; the dependent variable is natural logarithm of efficiency scores from the pooled output-oriented DEA; Log refers to natural log; the total number of observations is 120. The variance of random error is estimated at 0.111.
